# Mechanisms and Consequences of Defective Efferocytosis in Atherosclerosis

**DOI:** 10.3389/fcvm.2017.00086

**Published:** 2018-01-08

**Authors:** Arif Yurdagul, Amanda C. Doran, Bishuang Cai, Gabrielle Fredman, Ira A. Tabas

**Affiliations:** ^1^Department of Medicine, Columbia University, New York, NY, United States; ^2^Department of Pathology and Cell Biology, Columbia University, New York, NY, United States; ^3^Department of Physiology, Columbia University, New York, NY, United States; ^4^Department of Molecular and Cellular Physiology, Albany Medical College, Albany, NY, United States

**Keywords:** efferocytosis, atherosclerosis, inflammation resolution, macrophages, post-apoptotic necrosis

## Abstract

Efficient clearance of apoptotic cells, termed efferocytosis, critically regulates normal homeostasis whereas defective uptake of apoptotic cells results in chronic and non-resolving inflammatory diseases, such as advanced atherosclerosis. Monocyte-derived macrophages recruited into developing atherosclerotic lesions initially display efficient efferocytosis and temper inflammatory responses, processes that restrict plaque progression. However, during the course of plaque development, macrophages undergo cellular reprogramming that reduces efferocytic capacity, which results in post-apoptotic necrosis of apoptotic cells and inflammation. Furthermore, defective efferocytosis in advanced atherosclerosis is a major driver of necrotic core formation, which can trigger plaque rupture and acute thrombotic cardiovascular events. In this review, we discuss the molecular and cellular mechanisms that regulate efferocytosis, how efferocytosis promotes the resolution of inflammation, and how defective efferocytosis leads to the formation of clinically dangerous atherosclerotic plaques.

Efficient clearance of apoptotic cells, termed “efferocytosis,” is an ancient process that evolved to allow organ development, maintain homeostasis, prevent autoimmune disease, and resolve inflammatory insults (1). When efferocytosis functions efficiently, apoptotic cells are cleared before they become necrotic, anti-inflammatory cytokines and pro-resolving lipid mediators are secreted, and the release of immunogenic antigens is prevented. However, when efferocytosis becomes defective, uncleared apoptotic cells undergo post-apoptotic necrosis and release tissue-degrading enzymes, immunogenic epitopes, and pro-inflammatory mediators. Genetically modified mice show that impaired efferocytosis often develop autoimmune or chronic inflammatory diseases (2). Accordingly, there is substantial interest in understanding how efferocytosis becomes defective in chronic inflammatory diseases, such as atherosclerosis. This review will highlight the processes associated with efferocytosis and how these become dysregulated during atherosclerosis.

## Finding and Binding Apoptotic Cells

Despite the fact that the macrophage population in most organs and tissues are relatively low compared with other non-immune cells, apoptotic cells are rarely detected in tissues where high levels of cellular turnover are known to occur, indicating that macrophages rapidly mobilize to areas of cell death to expeditiously remove apoptotic corpses (3). Macrophage migration toward apoptotic cells is guided by chemotactic factors secreted by dying cells either actively in an executioner caspase-dependent mechanism or passively released during self-demise. This class of mediators, known as “find-me” signals, includes the classic chemokine CX3CL1, the lipids sphingosine 1-phosphate and lysophosphatidylcholine, and the nucleotides ATP and UTP (4–7).

**Figure 1:**
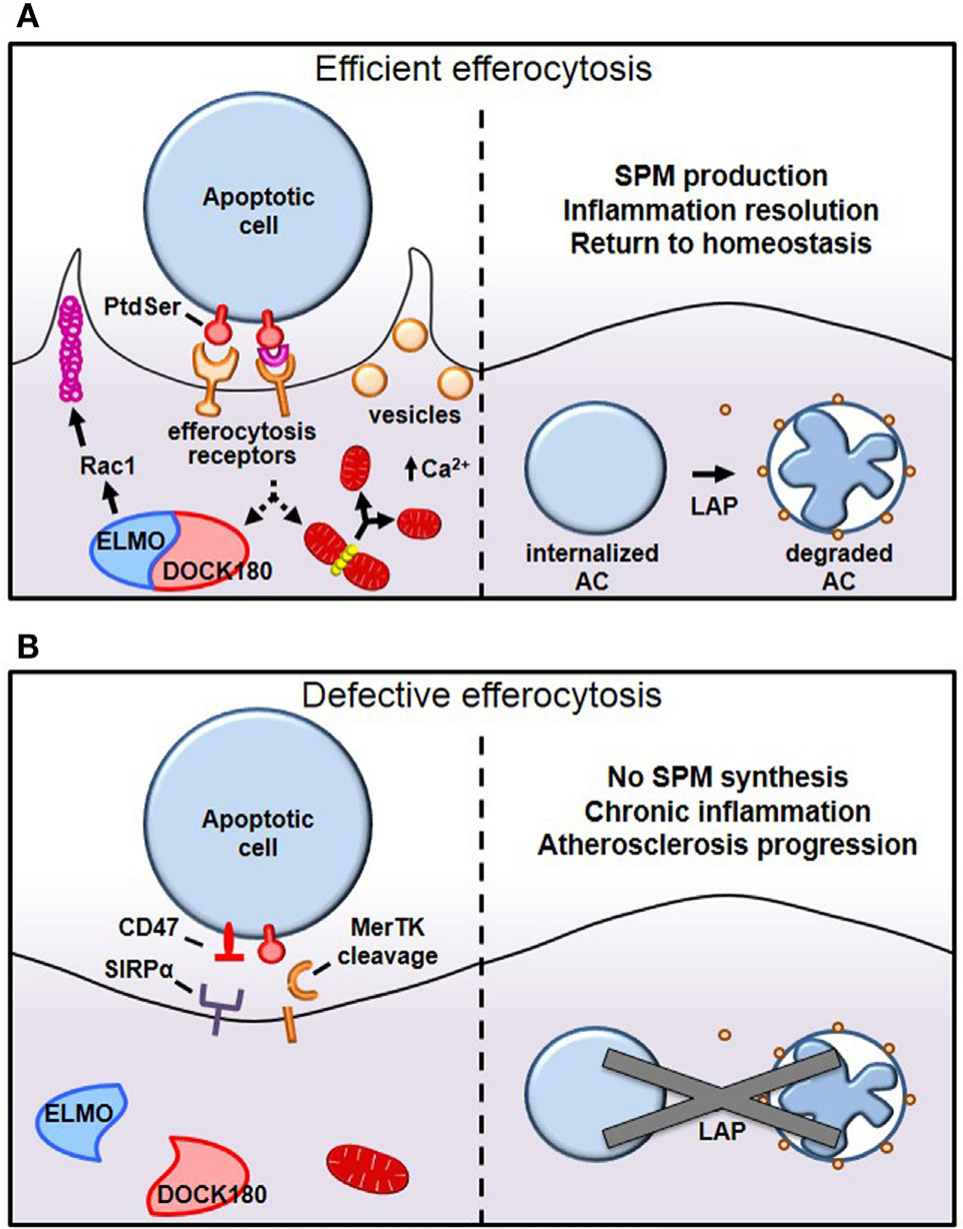
Mechanisms of efferocytosis. **(A)** Macrophages interact with phosphatidylserine (PtdSer) externalized on apoptotic cells either directly or indirectly, through bridging molecules. Many PtdSer receptors stimulate ELMO–DOCK180 interactions to activate Rac1 and polymerize actin around the phagosome. Simultaneously, macrophages trigger Drp1-mediated mitochondrial fission to drive calcium-dependent vesicular trafficking. Once internalized, autophagic machinery is used to conjugate lipids to LC3 bound to phagosomes, which drives phagolysosomal fusion and subsequent apoptotic cell degradation. **(B)** In pathological settings such as advanced atherosclerosis, one or more of these processes can become defective, leading to inefficient clearance of apoptotic cells and subsequent necrosis and inflammation. For example, in advanced atherosclerosis, apoptotic cells can inappropriately express the “don’t eat-me” signal CD47, or cell-surface receptors can get proteolytically cleaved, such as with MerTK.

After having navigated tissues to arrive at apoptotic-rich areas, macrophages employ a panoply of receptors that bind either directly or indirectly, *via* bridging molecules, to “eat-me” signals displayed on the surface of apoptotic cells (Figure 1) (8). While several “eat-me” signals have been identified, including changes in glycosylation at the cell surface or exposure of calreticulin or ICAM-1 epitopes, externalized phosphatidylserine (PtdSer) on apoptotic cells remains the most characterized (9, 10). Macrophages bind PtdSer directly through stabilin-1, stabilin-2, the GPCR brain angiogenesis inhibitor 1 (BAI1), or through the T-cell immunoglobulin and mucin domain family of receptors Tim-1, Tim-3, and Tim-4 (11–14). Alternatively, macrophages may utilize the Tyro3–Axl–Mer (TAM) family of tyrosine kinase receptors, integrins αVβ3 and αVβ5, or CD36 to bind PtdSer indirectly through bridging molecules that interact with PtdSer (3). Gas6 and Protein S bind to TAM receptors, whereas thrombospondin-1 or MFG–E8 link PtdSer to CD36 or integrins αVβ3 and αVβ5, respectively. Some of the PtdSer-relevant receptors have well-characterized signaling capabilities, e.g., MerTK, BAI1, and integrins, while others may function primarily as tethering and adhesion molecules, e.g., the Tim family of receptors and CD36.

Tethering and internalization are two separate but intimately linked events and operate first through interaction with weak and low avidity “eat-me” signals to ensure adhesion, followed by stereospecific interaction of externalized PtdSer to PtdSer receptors to drive cytoskeletal reorganization around the apoptotic cell. While externalized PtdSer on apoptotic cells binds to receptors on macrophages to mediate tethering, this process alone is insufficient to trigger internalization (15). However, coupling of PtdSer/receptor interaction with other receptors trigger the switch from adhesion to internalization, a process referred to as “tether and tickle” (16). Live cells may also express PtdSer and yet are spared from efferocytosis, primarily because live cells present the “don’t eat-me” signals CD31 and CD47 on their cell surface, which actively suppresses efferocytosis. CD31 is expressed on viable cells and cues repulsion or detachment when making homophilic interactions in trans with efferocytes (17). Interestingly, CD31 on macrophages may interact with apoptotic cells using the extracellular matrix protein fibronectin as a bridging molecule (18). When this occurs, integrin α5β1 becomes activated and subsequently promotes phagocytosis of apoptotic cells (18). Therefore, selective CD31 interactions not only prevents accidental internalization of viable cells but may also actively promote efferocytosis (Figure 1B). The other major “don’t eat-me” signal, CD47, is expressed on live cells and interacts with cell-surface signal-regulatory protein α on macrophages to inactivate myosin assembly and thereby prevent cytoskeletal rearrangement around the phagosome (19).

Internalization of apoptotic cells requires macrophages to dynamically reorganize their actin cytoskeleton to drive F-actin formation around apoptotic cells, forming a so-called phagocytic cup, which then promotes mechanical retraction of the phagosome into the cell (Figure 1A) (20). Since the Rho-family of small GTPases, Rac1, Cdc42, and RhoA rearrange the cytoskeleton to mechanically drive migration of cells, it is not surprising that they are also involved in mechanically internalizing phagosomes containing apoptotic cells. Using Forster resonance energy transfer biosensors, it was discovered that these small GTPase family members work in a temporally regulated fashion in which Rac1 and Cdc42 are activated early to facilitate phagocytic cup formation through actin polymerization followed by Rho activation, which drives mechanical retraction and phagosome internalization (21). Constitutive activation of Rac1 may decrease phagocytosis, because Rac1 must be rapidly inactivated to permit engulfment (21). However, when RhoA effectors are inhibited, to tilt the Rac1/RhoA axis toward Rac1 activation, uncontrolled phagocytosis occurs (22). A shared Rac1 activation pathway that is conserved among several of the apoptotic cell receptors involves association with the adaptor protein ELMO to the RacGEF DOCK180 (23). This ELMO/DOCK180 complex then activates Rac1 to initiate phagocytic cup formation, which then leads to apoptotic cell internalization (24). Accordingly, inhibition of ELMO/DOCK180 signaling prevents efferocytosis (13, 24).

While cytoskeletal remodeling is required for apoptotic cell internalization, the role of membrane trafficking in efferocytosis becomes astoundingly evident given that cells internalize ~50% of their entire surface, yet, plasma membrane surface area does not change as a result of phagocytosis (25). This finding suggests that internal membranes are rapidly recruited to the cell surface to complete closure of the phagosome and to replenish cell membranes utilized during efferocytosis. This concept can be demonstrated in tetanus or botulinum B toxin-microinjected cells, which show defective exocytosis and reduced membrane delivery to incoming phagosomes (26). New work has shown that these critical vesicular trafficking events require efferocytes to undergo Drp1-dependent mitochondrial fission (27). Mitochondrial fission causes endoplasmic reticulum calcium to be released into the cytosol rather than into the mitochondria, and this increase in cytosolic calcium then drives vesicular trafficking (27). Importantly, Drp1-deficient macrophages, which cannot undergo mitochondrial fission upon encountering apoptotic cells, are unable to move vesicles to the site of apoptotic cell attachment, which significantly delays both initial apoptotic cell phagosome sealing and, more notably, compromises the ability of the efferocytes to take up a second apoptotic cell (27).

## AC Corpse Degradation

Once apoptotic cells have been internalized, certain autophagy-related proteins are recruited to conjugate LC3-family proteins to lipids at the phagosome membrane, a process called LC3-associated phagocytosis (LAP) (Figure 1A) (28). LAP promotes phagosome fusion to lysosomes to drive hydrolytic degradation of apoptotic cell constituents (28). Importantly, defects in LC3 conjugation to phagosomal membranes delay or even prevent phagosome fusion with lysosomes, resulting in failure to acidify the phagosome and an inability to degrade apoptotic cells (29). After apoptotic cells are degraded in phagolysosomes, macrophages become overloaded with macromolecular constituents and therefore have evolved elegant mechanisms to either use or efflux this cargo. For instance, the burden of cholesterol from degraded apoptotic cells activates members of the peroxisome proliferator-activated receptor (PPAR) and liver X receptor (LXR) families of nuclear receptors and drive ABCA1 and ABCG1 expression, which mediate cholesterol efflux from the cells (30). Furthermore, PPARγ and LXR agonists further enhance efferocytosis (31, 32). To handle the large amount of chromosomal DNA derived from degraded apoptotic cells, macrophage lysosomes contain DNase II that cleaves this phagocytosed DNA. Macrophages lacking DNase II accumulate undigested DNA fragments, and mice lacking DNase II exhibit polyarthritis, an autoimmune disease similar to rheumatoid arthritis in humans (33).

## Atherosclerosis

Although the last several decades have seen significant medical advances in the diagnosis and treatment of cardiovascular disease, atherosclerosis remains the major cause of morbidity and mortality worldwide (34). Atherosclerosis begins when circulating apolipoprotein B-containing lipoproteins accumulate in focal areas in the subendothelium matrix of medium-sized and large arteries (35). These subendothelial lipoproteins, particularly after oxidation, generate an inflammatory stimulus that drives leukocyte influx into the vessel wall (36–39). Primary among these infiltrating cells are monocyte-derived macrophages, which internalize cholesterol-rich lipoproteins and give rise to foam cells. Foam cells secrete extracellular matrix that further promotes lipoprotein retention as well as pro-inflammatory cytokines that augment the recruitment of additional monocytes, T cells, and neutrophils. In the face of persistent inflammatory stimuli and other cytotoxic factors, many lesional cells become apoptotic. Early on, apoptotic cells are efficiently cleared by neighboring macrophages in an attempt to limit overall lesion cellularity (40). However, efferocytosis can fail as plaques progress, leading to the accumulation of secondarily necrotic cells and the formation of a highly inflammatory “necrotic core” (41–43). Large necrotic cores are a hallmark of advanced atherosclerotic disease and have been associated with the types of atherosclerotic plaque that give rise to heart attack and stroke (44, 45). Therefore, the efficient clearance of dead and dying cells plays a key role in preventing the development of clinically significant atherosclerotic plaques.

## Mechanisms of Impaired Efferocytosis in Atherosclerosis

Why does efferocytosis fail in advanced atherosclerosis? Because efferocytosis is a high-capacity process, it is unlikely that overwhelming lesional apoptosis is the primary cause. Rather, efferocytosis itself becomes defective and/or lesional apoptotic cells become poor substrates for efferocytosis. As an example of the latter, CD47 expression is significantly increased in human atherosclerotic plaque cells, presumably *via* a TNFα-dependent mechanism, and for the reasons explained earlier (19), these cells are poorly internalized by lesional efferocytes (46) (Figure 1B). In keeping with this concept, administration of CD47-blocking antibodies to atheroprone mice led to improved lesional efferocytosis and smaller necrotic cores. Other findings suggest that dead cells in lesions express lower amounts of the “eat-me” signal calreticulin (47). For example, *Apoe^−/−^* mice lacking *Cdkn2b* show decreased levels of calreticulin, and apoptotic bodies from these animals show resistance to being engulfed (47). When fed a Western diet, these mice have an increased overall lesion size as well as increased necrotic core size (47) (Table 1). Interestingly, human carriers of the cardiovascular risk allele at the chromosome 9p21 GWAS locus were found to have lower intraplaque expression of calreticulin, suggesting that defective efferocytosis may contribute to cardiovascular disease in these patients (48).

**Table 1:** Efferocytosis pathway molecules shown to have a causative role in atherosclerosis.

Molecule	Function	Animal model	Effect on aortic lesion area	Effect on necrotic core size	Other findings	Reference
MerTK	Receptor				↑ ACs	
					↓ *In situ* efferocytosis	
		MerTK^KD^ Apoe^−/−^ mice	No change	↑	↑ Lesional macrophages	(49)
		MerTK^−/−^ marrow → Ldlr^−/−^ mice	↑	↑	↓ *In situ* efferocytosis	(50)
		MerTK^CR^ Ldlr^−/−^ mice	No change	↓	↑ Collagen cap thickness	(51)
					↑ T regulatory cells	
					↑ Specialized pro-resolving mediators	

Lipoprotein receptor-related protein 1 (LRP1)	Receptor	Macrophage LRP1^−/−^ marrow → Ldlr^−/−^ mice Macrophage LRP1^−/−^ Apoe^−/−^ mice	↑	↑	↑ ACs ↓ *In situ* efferocytosis ↑ Lesional macrophages ↑ MMP9 levels	(52, 53)
			↑	↑	↑ ACs ↓ *In situ* efferocytosis ↑ Lesional macrophages	(54)

SR-B1	Receptor	SR-B1^−/−^ ApoE^−/−^ marrow → Apoe^−/−^ mice SR-B1^−/−^ marrow → Ldlr^−/−^ mice	↑	↑	↑ ACs ↓ *In situ* efferocytosis ↓ Lesional macrophages ↓ Collagen lesion area and cap thickness ↑ ACs ↓ *In situ* efferocytosis ↓ Lesional macrophages ↓ Collagen lesion area and cap thickness	(55)

Tim-1/Tim-4	Receptor	Ldlr^−/−^ treated with Tim-1 or Tim-4 blocking antibodies	↑	Not tested	↑ ACs ↓ *In situ* efferocytosis ↑ Lesional T cells	(56)

Mineralo-corticoid receptor	Non-efferocytosis nuclear receptor	Myeloid MRKO^−/−^ marrow → Ldlr^−/−^ mice	↓	↓	↓ ACs ↑ *In situ* efferocytosis ↓ Lesional macrophages ↓ Foam cell formation ↑ Collagen lesion area	(57)

MFG-E8	Bridging molecule	MFG-E8^−/−^ marrow → Ldlr^−/−^ mice	↑	↑	↑ ACs ↑ Collagen cap thickness	(58)

C1q	Bridging molecule	C1q^−/−^ Ldlr^−/−^	↑	Not tested	↑ ACs ↑ Lesional macrophages *In vitro*: incubating macrophage cell line with C1q enhances C19-mediated efferocytosis	(59)
						(60)

Transglutaminase 2 (TG2)	Bridging molecule	TG2^−/−^ marrow → Ldlr^−/−^ mice	↑	↑	↑ Lesional macrophages *In vitro*: TG2^−/−^ macrophages have decreased efferocytosis	(61)

Gas6	Bridging molecule	Gas6^−/−^ Apoe^−/−^ mice	No change	↓	↑ Collagen content	(62)

CX3CL1	Find-me signal	CX3CL1^−/−^ Apoe^−/−^ mice CX3CL1^−/−^ Ldlr^−/−^ mice	Inconsistent change at aortic root, ↓ at brachiocephalic artery ↓At aortic root and brachiocephalic artery	Not tested Not tested	↓ Lesional macrophages ↓ Lesional macrophages	(63)

Fas/Fas ligand	Mediates find-me signaling	Fas^−/−^ Apoe^−/−^ mice Gld Apoe^−/−^ mice	↑ ↑	Not tested Not tested	↑ ACs ↑ ACs ↑ Lesional macrophages ↑ T cells ↓ *In situ* efferocytosis in lymph tissue	(64)
						(65)

Calreticulin	Eat-me signal	*Cdkn2b^−/−^ Apoe^−/−^* mice have low levels of calreticulin	↑	↑	↓ Collagen content and cap thickness ↓ *In situ* efferocytosis by semi-quantitative measure *In vitro*: loss of CKDN2B leads to ↓ efferocytosis	(47)

CD47	Don’t eat-me signal	*Apoe^−/−^* mice treated with CD47-blocking antibody	↓	↓	↓ ACs ↑ *In situ* efferocytosis	(46)

miR-21	MicroRNA	miR21^−/−^ marrow → Ldlr^−/−^ mice	↑	↑	↑ ACs ↓ Lesional macrophages ↓ Collagen cap area with no change in lesional collagen content	(66)
					*In vitro*: miR21^−/−^ macrophages have ↓MerTK and ↓ efferocytosis	(66, 67)

Efferocytosis may also be compromised by competition for apoptotic cell binding. As atherosclerosis progresses, lesions continue to accumulate lipids and ROS, leading to increased levels of oxidized phospholipids. These lipids can bind to efferocytosis receptors and may compete for apoptotic cell recognition (68). Similarly, autoantibodies against oxLDL and other oxidized phospholipids are able to bind to and potentially mask “eat-me” ligands on the surface of dying cells in the lesions (69, 70). Further, oxLDL increases the expression of and signaling through toll-like receptor 4 (TLR4), leading to increased secretion of the pro-atherogenic cytokines TNFα and IL-1β while reducing the anti-inflammatory cytokines TGFβ and IL-10 (71). This pro-inflammatory environment impairs efferocytosis by reducing the expression of various key efferocytosis molecules, as discussed below, and promotes increased lipid uptake at the expense of phagocytosis (72).

Finally, accumulating experimental evidence has demonstrated that the expression and function of efferocytosis receptors and their bridging molecules are deficient in advanced atherosclerosis. One such example is MerTK and its associated bridging molecule, Gas6. MerTK is expressed by macrophages in both murine and human plaques (50, 51). As lesions progress, MerTK levels on the macrophage surface decline, and this decrease is associated with cleavage of the receptor by the metalloproteinase ADAM17 (Figure 1B). Multiple athero-relevant inflammatory stimuli have been shown to promote ADAM17 activity and MerTK cleavage (73–75). Indeed, levels of the soluble fragment of the receptor (solMer) accumulate within the aortas of mice and in human carotid plaques (51). *In vitro*, solMer has been shown to inhibit efferocytosis by competing with Gas6, suggesting that this may amplify the deleterious effects on efferocytosis (73). Using a genetically engineered mouse in which the cleavage domain of MerTK has been rendered resistant, Cai and colleagues demonstrated that cleavage of the MerTK receptor is a causal factor in the development of necrotic cores in atherosclerotic lesions (51). Consistent with this protective role for MerTK activity in atherosclerosis, loss of MerTK, either by genetic deletion or through models in which MerTK has been replaced by a version with an inactive kinase domain, results in increased lesion size and larger necrotic cores (49, 50) (Table 1). Interestingly, deletion of the related TAM family member, Axl, in bone marrow cells of *Ldlr^−/−^* mice did not affect lesional efferocytosis or plaque necrosis in advanced atherosclerosis (76).

Low-density lipoprotein receptor-related protein 1 (LRP1) is a receptor that is activated by calreticulin on the surface of apoptotic cells (77). The macrophage receptor LRP1 can also be downregulated in response to TLR4 signaling and inactivated by ADAM17-mediated proteolytic cleavage (78, 79). Several studies have demonstrated that the loss of *Lrp1* in macrophages or in bone marrow cells leads to increased lesion area and necrotic core size in an *Apoe^−/−^* mice (52–54) (Table 1). A particular bridging molecule that is reduced in atherosclerotic lesions is milk fat globule-epidermal growth factor 8 (MFG-E8), which functions to tether apoptotic cells to efferocytes by interacting with αVβ3 integrins and the transglutaminase 2 (TG2) co-receptor on phagocytes (80, 81). MFG-E8 is expressed in atherosclerotic plaques, but its expression declines in advanced plaque, potentially owing to downregulation by inflammatory stimuli (82). In an *Ldlr^−/−^* mouse model lacking *Mfge8* in bone marrow cells, larger plaque area and necrotic cores were observed (58). In addition, *Ldlr^−/−^* mice lacking TG2 in bone marrow also show increased plaque area and necrotic core size (81). Another bridging molecule, complement component 1q (C1q), has also been shown to be important in atherosclerosis. *In vitro*, macrophages can produce large amounts of C1q, which promotes both cell survival and efferocytosis (60). Loss of C1q from *Ldlr^−/−^* mice led to larger lesion area and an increase in apoptotic cells, consistent with defective apoptotic cell clearance (59). As another possible mechanism for defective efferocytosis, the pro-inflammatory molecule high-mobility group box 1 (HMGB1) is increased in human and murine atherosclerosis (83, 84), and the secreted form has been shown to interact with integrin αVβ3 and PtdSer to block efferocytosis (85, 86). *Apoe^−/−^* mice administered an anti-HMGB1 antibody developed smaller atherosclerotic plaques, although necrotic core size was not reported (84). Silencing of HMGB1 in peritoneal macrophages *in vitro* leads to increased efferocytosis, and partially rescues the efferocytosis defect observed in SR-B1*^−/−^* macrophages (55).

Recently, microRNAs have been found to have a novel role in the regulation of efferocytosis. Das and colleagues found that macrophages undergoing efferocytosis increase their expression of miR-21 in a TLR4-dependent manner *in vitro* (67). Further, when treated with an miR-21 mimetic *in vitro*, the rate of efferocytosis by bone marrow-derived macrophages increased (67). Transplantation of *miR21^−/−^* marrow into *Ldlr^−/−^* mice increased plaque area and necrotic core size. One study reported that loss of miR-21 in macrophages decreases MerTK expression, providing a mechanism for the increased necrotic core size in these mice (66). Additional work is necessary to determine the mechanism by which miR-21 regulates MerTK expression. Another miR, miR-33, is also known to regulate the outcome of atherosclerosis. Murine primary macrophages treated with anti-miR-33 enhanced efferocytosis *in vitro* and treatment of *Ldlr^−/−^* mice with anti-miR-33 decreased necrotic cores compared with the anti-miR control (87). Together, these results suggest that specific miRs play important roles in regulating efferocytosis in atherosclerosis.

## Efferocytosis and Inflammation Resolution

To successfully terminate an inflammatory process, the active process of inflammation resolution is required (88). This process is mediated by various endogenous molecules, including bioactive lipids such as lipoxins, resolvins, protectins, and maresins, which are often referred to as specialized pro-resolving mediators (SPMs); proteins such as annexin A1 and interleukin-10; and gasses such as hydrogen sulfide (88). When the resolution program fails inflammation persists, and this mechanism is now understood to be an underlying factor in the pathogenesis of many chronic inflammatory diseases, including atherosclerosis (89). Emerging evidence has defined an important role for resolution and SPMs in both murine and human atherosclerotic disease (89–91). Gene variants encoding proteins and enzymes necessary for SPM biosynthesis, including 5-lipoxygenase (5-LOX), have been associated with atherosclerosis, stroke, and myocardial infarction in selected populations (92–96). Patients with coronary artery disease have lower plasma SPMs than do healthy patients (97), and one SPM, aspirin-triggered lipoxin A_4_, was found to be significantly associated with peripheral and coronary atherosclerosis in humans even after correction for age, sex, and C-reactive protein levels (98). A recent paper showed that stable regions of human atherosclerotic plaque have a higher SPM:leukotriene ratio when compared with more advanced, vulnerable regions having larger necrotic cores and thinner collagen caps (99). Similarly, early murine lesions from Western diet-fed *Ldlr^−/−^* mice had a higher SPM:leukotriene ratio when compared with advanced lesions (90, 99). In several mouse models of atherosclerosis, treatment of mice with various pro-resolving ligands including annexin 1, Ac2-26, IL-10, resolvin D1 (RvD1), resolvin D2, or maresin 1 decreased lesional necrosis, suggesting improvements in efferocytosis by lesional phagocytes (90, 99, 100–102).

Efferocytosis plays a major mechanistic role in the resolution of inflammation. First, expeditious clearance of dead cells prevents their secondary necrosis. Second, the act of efferocytosis itself triggers several different anti-inflammatory and pro-resolving signaling pathways. Engagement and activation of the TAM family of efferocytosis receptors, including MerTK and Axl, induces the expression of suppressor of cytokine signaling-1 and 3 (SOCS-1 and 3), leading to the inhibition of signaling pathways triggered by cytokines and toll-like receptor ligands (103, 104). Efferocytosis has also been shown to actively increase the secretion of anti-inflammatory cytokines, including TGF-β and IL-10, and decreased secretion of pro-inflammatory cytokines, such as TNF-α, IL-1β, and IL-8 (105, 106). Further, uptake of apoptotic cells enhances the synthesis of SPMs, while concomitantly reducing the production of pro-inflammatory leukotrienes (107, 108). Recently, a specific mechanistic link between the efferocytosis receptor MerTK and SPM production was revealed (108). In response to engagement of the MerTK receptor, the key biosynthetic enzyme 5-LOX translocates from the nucleus to the cytoplasm, where it drives the production of the pro-resolving mediator lipoxin A_4_. When MerTK is inactivated either genetically or proteolytically, 5-LOX is restricted to the nuclear membrane, where it instead favors the production of the pro-inflammatory leukotriene B_4_ (108). Mice whose myeloid cells express a cleavage-resistant variant of MerTK (MerTK^CR^ mice) have higher rates of efferocytosis than their wild type counterparts, and macrophages from these mice demonstrate enhanced production of LXA_4_ and RvD1. In addition, when *Ldlr^−/−^* mice are transplanted with bone marrow from MerTK^CR^ mice and fed an atherogenic diet for 16 weeks, the aortas contained an increased SPM:leukotriene ratio (51). The process of resolution in atherosclerosis can also enhance efferocytosis. A recent study showed that administration of RvD1 to Western diet-fed *Ldlr^−/−^* mice significantly increased the SPM:leukotriene ratio, while also decreasing plaque necrosis and enhancing lesional efferocytosis (99). These studies suggest a positive feedback cycle between resolution and efferocytosis, which, if interrupted, can lead to an amplification loop of inflammation and necrosis that promotes advanced atherosclerotic plaque progression.

## Summary and Conclusion

Defective clearance of apoptotic cells in atherosclerotic lesions drives post-apoptotic necrosis of lesional cells and inflammation triggered by the release of cellular debris from these necrotic cells (2). Moreover, active cell signaling programs of inflammation suppression and inflammation resolution in efferocytes are often lost when apoptotic cells are not properly cleared (89). As a result, defective efferocytosis can transform stable, asymptomatic atherosclerotic lesions into necrotic, inflammatory, and non-resolving plaques that are prone to rupture (41). Although we do not know for certain why efferocytosis fails in advanced atherosclerosis, studies thus far suggest complementary mechanisms that involve both poor recognition of lesional apoptotic cells, e.g., due to inappropriate expression of CD47, coupled with defects in the efferocytes themselves; e.g., due to proteolytically cleavage of MerTK (46, 51, 108).

How might this knowledge suggest new types of therapies to prevent atherothrombotic vascular disease? Therapies that lower LDL in the blood are the mainstay of therapy to prevent atherosclerotic disease, and there is reason to posit that this type of therapy can indirectly prevent processes in plaques, such as inflammation and oxidative stress, that may ultimately contribute to defective efferocytosis. However, to the extent that many subjects at risk are not able to lower their LDL to a level low enough to completely suppress atherosclerotic disease, there is a place for complementary therapies (109). For example, recent success of the CANTOS trial demonstrated that lowering inflammation, through administering an anti-IL-1β antibody, successfully reduced recurrent cardiovascular events independently of lipid lowering (110). One type of new approach that may successfully target defective efferocytosis is antibodies that block CD47. However, anti-CD47 antibodies also causes anemia owing to inappropriate clearance of red blood cells (46, 111, 112). Another type of approach is to enhance the function of efferocytes themselves by preventing proteolysis of efferocytosis receptors, e.g., by blocking ADAM17-mediated cleavage of MerTK, or by enhancing the ability of efferocytes to clear multiple apoptotic cells, e.g., by boosting the mitochondrial fission-calcium mechanism that enables macrophages to efficiently ingest secondarily encountered apoptotic cells (27, 51, 108). Yet another approach would be tilting the SPM:leukotriene ratio to favor SPM production, such as through the administration of RVD1, which has been shown to enhance macrophage–apoptotic cell interactions and to increase lesional efferocytosis (51, 99). Finally, glucocorticoids generate anti-inflammatory molecules and are therefore routinely used for managing inflammatory diseases. One such glucocorticoid product, annexin A1, enhances efferocytosis, resolves inflammation, and delays atherosclerosis in mice (113–115). Indeed, the combination enhancing efferocytosis while at the same time restoring resolution mediators in lesions may offer the most promising therapeutic strategy to combat atherosclerotic cardiovascular disease.

## Author Contributions

All authors contributed to drafting and editing the review. AY designed the graphic in Figure 1 and AD designed Table 1.

## Conflict of Interest Statement

The authors declare that the research was conducted in the absence of any commercial or financial relationships that could be construed as a potential conflict of interest.
